# Mitochondria-associated endoplasmic reticulum membranes as a therapeutic target for cardiovascular diseases

**DOI:** 10.3389/fphar.2024.1398381

**Published:** 2024-04-17

**Authors:** Yanqiu Ding, Nanyang Liu, Dawu Zhang, Lijun Guo, Qinghua Shang, Yicheng Liu, Gaocan Ren, Xiaochang Ma

**Affiliations:** ^1^ Cardiovascular Department, Xiyuan Hospital, China Academy of Chinese Medical Sciences, Beijing, China; ^2^ Graduate School, Beijing University of Chinese Medicine, Beijing, China; ^3^ Department of Geratology, Xiyuan Hospital, China Academy of Chinese Medical Sciences, Beijing, China; ^4^ State Key Laboratory of Traditional Chinese Medicine Syndrome, Xiyuan Hospital, China Academy of Chinese Medical Sciences, Beijing, China

**Keywords:** cardiovascular diseases, mitochondria-associated endoplasmic reticulum membrane, therapeutic target, coronary atherosclerosis, myocardial ischemia

## Abstract

Cardiovascular diseases (CVDs) are currently the leading cause of death worldwide. In 2022, the CVDs contributed to 19.8 million deaths globally, accounting for one-third of all global deaths. With an aging population and changing lifestyles, CVDs pose a major threat to human health. Mitochondria-associated endoplasmic reticulum membranes (MAMs) are communication platforms between cellular organelles and regulate cellular physiological functions, including apoptosis, autophagy, and programmed necrosis. Further research has shown that MAMs play a critical role in the pathogenesis of CVDs, including myocardial ischemia and reperfusion injury, heart failure, pulmonary hypertension, and coronary atherosclerosis. This suggests that MAMs could be an important therapeutic target for managing CVDs. The goal of this study is to summarize the protein complex of MAMs, discuss its role in the pathological mechanisms of CVDs in terms of its functions such as Ca^2+^ transport, apoptotic signaling, and lipid metabolism, and suggest the possibility of MAMs as a potential therapeutic approach.

## 1 Introduction

Cardiovascular diseases (CVDs), such as cardiomyopathy, heart failure (HF), hypertension, and atherosclerosis, are a major cause of mortality worldwide (Amini et al., 2021; Ren et al., 2021). In 2022 alone, it caused 19.8 million deaths worldwide, accounting for 33% of global deaths. It is noteworthy that 34% of these deaths occur before the age of 70 (Mensah et al., 2023). Given the aging population and lifestyle changes, this disease poses a significant challenge to human health. Due to the severe lethality of the disease, the study of its pathogenesis has been the focus of pharmacological attention.

Mitochondria are considered to be the primary site for aerobic respiration and energy production. Additionally, they are key regulators of reactive oxygen species (ROS) production, inflammation, metabolism, and cell death (He et al., 2022). It has been demonstrated through numerous studies that mitochondria play a crucial role in CVDs. For example, recent research has shown that mitochondrial energy metabolism and translation are crucial factors in cardiac regeneration (Li et al., 2023; Gao et al., 2023). A recent study found that the promotion of MCM8 and E3 ubiquitin ligase tripartite motif containing 21 (TRIM21) by nitric oxide can mediate mitochondrial autophagy, which in turn helps to maintain normal coronary artery function (Lin et al., 2023). The endoplasmic reticulum (ER) is a crucial site for protein synthesis and folding. It is an interconnected network of different morphologies that extend throughout the cytoplasm, forming abundant contacts with other organelles. Additionally, the ER plays a role in regulating Ca^2+^ and lipid metabolism (Parkkinen et al., 2023; Zheng et al., 2023). Maintaining ER protein homeostasis is crucial for preserving cellular secretory function. It is important to note that long-term ER stress can result in cellular defects and CVDs (Ren et al., 2021). Additionally, interactions between organelles are essential for organelle function and overall cellular homeostasis (Gatta and Levine, 2017). Mitochondria-associated endoplasmic reticulum membranes (MAMs) are considered to be communication platforms that regulate cellular physiological functions between mitochondria and endoplasmic reticulum organelles in both directions, which may be believed to influence cell fate (Wang et al., 2021; Zhang et al., 2023; Yang and Luan, 2023). According to the report, dysfunctions in the structure and function of MAMs can lead to the development of various pathological conditions and diseases in the body, including CVDs (Missiroli et al., 2018).

Recently, there has been an increasing number of studies investigating the role of MAM proteins in CVDs. For instance, one study discovered that FUN14 domain-containing 1 (FUNDC1) facilitates the formation of MAMs, leading to elevated expression of vascular endothelial growth factor R2 and ultimately promoting angiogenesis (Wang et al., 2021). The transmembrane protein 215 (TMEM215) is a transmembrane protein located on the endoplasmic reticulum that inhibits BIK (BCL-2 interacting killer)-regulated ER-to-mitochondria Ca^2+^ inward flow, thereby preventing endothelial cell apoptosis during vascular recovery (Zhang et al., 2023). Lon protease 1 (LonP1), a novel protein localized to MAMs, is involved in regulating cardiomyocyte function and maintaining normal cardiac function (Li et al., 2023). The purpose of this study is to describe the structure and function of MAMs, elaborate on their mechanism of action in CVDs, and discuss interventions related to the prevention or treatment of CVDs.

## 2 The components of mitochondria-associated endoplasmic reticulum membranes

MAMs are regions where the outer mitochondrial membrane (OMM) and certain areas of the ER membrane overlap without membrane fusion. It was first isolated from rat liver in the 1990s (Vance, 1990). Under electron microscopy, the distance between the OMM and the ER was maintained at 10–25 nm (Csordás et al., 2006). Proteomic analysis revealed that MAMs contain over 1000 enriched proteins across various species and tissues (Poston et al., 2013; Wang et al., 2018). Studies have shown that proteins in MAMs exhibit dynamic behavior, with some being transient while others are persistent (Janikiewicz et al., 2018). The biological functions of MAMs are carried out through mitochondria-endoplasmic reticulum contacts (MERCs) (Barazzuol et al., 2021), which are protein complexes that perform a variety of functions such as Ca^2+^ signaling, lipid metabolism, oxidative stress, autophagy, apoptosis, etc (Zhang et al., 2023; Yang and Luan, 2023). Loss of MERCs homeostasis is manifested by Ca^2+^ overload, accumulation of unfolded or misfolded proteins, etc., which in turn leads to cell degeneration and pathology.

The proteins of MERCs were classified into three categories based on mass spectrometry analysis. These categories include 1) MAMs-resident proteins, which are present only on MAMs; 2) MAMs-enriched proteins, which are also present in other regions of the cell; and 3) MAMs-associated proteins, which are temporarily located on MAMs (Poston et al., 2013). Among the major proteins associated with the cardiovascular system are the inositol 1,4,5-trisphosphate receptor (IP3R), glucose-regulated protein 75 (GRP75), voltage-dependent anion channel 1 (VDAC1), sigma 1 receptor (Sig1R), mitofusin 1 and 2 (MFN1/2), vesicle-associated membrane protein-associated protein B (VAPB), protein tyrosine phosphatase interacting protein 51 (PTPIP51), fission protein 1 (Fis1), B-cell receptor-associated protein 31 (BAP31), phosphofurin acidic cluster sorting protein 2 (PACS2), etc. Further, the proteins can be classified according to their functions, including Ca^2+^ regulatory proteins, redox regulatory proteins, lipid synthesis and transport proteins, autophagy-related proteins, etc (Li et al., 2022). The focus of this study is on the mechanism of these proteins in cardiovascular homeostasis and metabolic mechanisms associated with CVDs.

### 2.1 IP3R1-GRP75-VDAC1 complex

The IP3R1 protein is located on the surface of the ER and facilitates the transfer of Ca^2+^ from the ER to the cytoplasm. The VDAC1 protein is an ion channel located on the OMM that regulates metabolites and ions within the mitochondria. Additionally, it is capable of promoting Ca^2+^ into the mitochondria. The GRP75 protein acts as a bridge between IP3R and VDAC, forming the intact protein complex and facilitating the interaction between IP3R and VDAC (Basso et al., 2020). The IP3R1-GRP75-VDAC1 complex has been found to play a significant role in regulating the cardiovascular system. Specifically, IP3R1 has been identified as a central player in the development of cardiac hypertrophy (Nakayama et al., 2010; Garcia et al., 2017), while IP3R1 in vascular smooth muscle cells (VSMCs) has been shown to contribute to peripheral vasoconstriction during HF (Dridi et al., 2022). Additionally, one study reported that a reduction in VDAC1 during myocardial ischemic injury resulted in a decrease in the area of myocardial infarction (Feng et al., 2019).

### 2.2 BAP31-Fis1 complex

BAP31 is a protein located in the ER, while Fis1 is in the OMM. In the process of apoptosis, procaspase-8 is recruited to the BAP31-Fis1 platform, which leads to the processing of BAP31 and the formation of p20BAP31. This signaling pathway activates mitochondria for apoptosis by releasing Ca^2+^ from the ER (Iwasawa et al., 2011). Furthermore, it is noted that PACS2 proteins play a critical role in regulating MAMs. If PACS2 protein is depleted, BAP31 is cleaved into p20, which leads to the release of Ca^2+^ from the ER to the mitochondria. This, in turn, triggers the recruitment of dynamin-related protein 1 (Drp1) to the mitochondria to activate mitochondrial fission, ultimately resulting in the fragmentation of the mitochondrial network (Li et al., 2020). During prolonged hypobaric hypoxia, it has been observed that the proteins PACS2 related to MAMs and mitochondrial autophagy are downregulated. Studies have shown that knockdown and knock-in of PACS2 can cause cardiomyocyte injury, exacerbation, and recovery of right heart dysfunction (Yang et al., 2023). Additionally, research has demonstrated that stabilizing BAP31 in septic cardiomyopathy can help protect cardiac function (Zhang et al., 2020). Moreover, Zhou and colleagues have found that the desumoylation of endothelial cell Fis1 may help maintain mitochondria, which could potentially prevent hypoxic pulmonary hypertension (Zhou et al., 2023).

### 2.3 VAPB-PTPIP51 complex

VAPB is a protein found in the ER that is responsible for vesicle trafficking and the unfolded protein response. PTPIP51 is located in the OMM and regulates cell development and tumorigenesis (Vinay Kumar et al., 2014; Gómez-Suaga et al., 2019). It has been suggested that VAPB forms a complex with PTPIP51 to maintain the structure of MAMs and regulate the transport of Ca^2+^ (Gómez-Suaga et al., 2019). Research has indicated that the VAPBP56S mutation results in a heightened attraction to PTPIP51, which facilitates the transfer of Ca^2+^ from the ER to the mitochondria. The reduction of Ca^2+^ transport occurs when a gene is suppressed (De Vos et al., 2012). Disruption of the structure of MAMs affects Ca^2+^ conductance and ATP synthesis (Paillusson et al., 2017). Furthermore, the VAPB-PTPIP51 complex’s function is influenced by α-synuclein (Gómez-Suaga et al., 2019) and TAR DNA-binding domain protein 43 (Stoica et al., 2014). The transfer of phosphatidylserine (PS) from the ER to the mitochondria is promoted by the interaction between ORP5/8 on the ER and PTPIP51 on the OMM. It has been observed that depletion of ORP5/8 leads to defects in mitochondrial morphology and respiratory function (Galmes et al., 2016). One study discovered that PTPIP51 was significantly upregulated in myocardial ischemia-reperfusion (I/R) (Qiao et al., 2017). Additionally, they found that specific knockdown of PTPIP51 reduced myocardial infarction size. According to a study, it was found that the expression of the VAPB-PTPIP51 complex was significantly reduced in a hypertensive mouse model (Liu T. et al., 2023).

### 2.4 MFN2-MFN1/2 complex

The protein MFN2, which is responsible for mitochondrial fusion, is present in both the OMM and the ER (de Brito and Scorrano, 2008). It forms hetero- or homodimers with MFN1/2 on the OMM to regulate the distance between organelles and coordinate the ER and mitochondrial dynamics (Naon et al., 2016; Filadi et al., 2018). However, there are conflicting findings in current studies. One study showed that when MFN2 was removed or silenced, the distance between the ER and mitochondria decreased, resulting in an increased transfer of inositol trisphosphate (IP3)-induced Ca^2+^ from the ER to mitochondria (Filadi et al., 2015). However, another study found that the acute removal of MFN2 resulted in a decrease in mitochondrial uptake of Ca^2+^ released from the ER (Naon et al., 2016). Moreover, it has been observed that the knockdown of MFN2 leads to mitochondrial swelling, degeneration, and increased oxidative damage, which ultimately results in apoptosis (Han et al., 2020).

The MFN2-MFN1/2 complex is involved in the regulation of the cardiovascular system. One study discovered that MFN2 expression was downregulated in myocardium hypertrophied compared to normal myocardium (Sun et al., 2019). Furthermore, in an animal study, the overexpression of MFN2 inhibited the formation of atherosclerosis (Guo et al., 2007). In a study conducted on mice, the researchers found that the absence of MFN1/2 in hearts prevented acute myocardial infarction (Hall et al., 2016).

## 3 The functions of mitochondria-associated endoplasmic reticulum membranes

MAMs are recognized as important functional regions within cells where multiple biological events occur, such as Ca^2+^ signaling, lipid metabolism, mitochondrial dynamics, apoptosis, etc (Giamogante et al., 2021; Li et al., 2022).

### 3.1 Ca^2+^ signaling

Ca^2+^ is known to play a crucial role in cell proliferation, growth, and death (Giorgi et al., 2018). The transduction of Ca^2+^ between the ER and mitochondria is a complex process that involves various protein complexes. The IP3R1-GRP75-VDAC1 complex is considered one of the most prominent regulatory pathways (Basso et al., 2020). The ER releases Ca^2+^ through IP3R1, resulting in the formation of a region of high Ca^2+^ concentration near the ER. Additionally, VDAC1, a Ca^2+^ uptake channel located on the OMM, connects to GRP75 through the cytoplasm. It is worth noting that neither overexpression nor deficiency of GRP75 has been observed to alter the contact distance between the ER and mitochondria. However, it has been found that deficiency of GRP75 reduces the Ca^2+^ uptake of mitochondria (Szabadkai et al., 2006; Honrath et al., 2017). This protein complex also serves as a molecular scaffold for other Ca^2+^ regulators. Studies have shown that pyruvate dehydrogenase kinases 4 (Thoudam et al., 2019), DJ-1 (Liu et al., 2019; Basso et al., 2020), and Sig1R (Su et al., 2016) interact with the ER protein chaperone binding immunoglobulin protein (BiP) to maintain the stability of the IP3R-GRP75-VDAC complex, thereby enhancing its Ca^2+^ regulatory function. Additionally, transient receptor potential melastatine 8 and ryanodine receptor (RyR) are also involved in Ca^2+^ regulation (Bidaux et al., 2018).

The function of the cardiovascular system is highly dependent on Ca^2+^ signaling. According to a study, the depletion of the protein kinase RNA-like endoplasmic reticulum kinase (PERK) in cells with diabetic cardiomyopathy resulted in decreased activity of calcineurin and RyR2 channels, which impaired intracellular Ca^2+^ accumulation (Liu et al., 2014). This may be responsible for ventricular arrhythmias in diabetic cardiomyopathy. Additionally, in cardiomyocytes, FUNDC1, located in the OMM, directly binds to IP3R2 and regulates Ca^2+^ release. Abnormal Ca^2+^ metabolism is associated with mitochondrial fission, which may lead to cardiac dysfunction and HF (Wu et al., 2017). Additionally, Ca^2+^ is known to play a crucial role in vasoconstriction and resistance in VSMCs, and transient receptor potential vanilloid (TRPV) 4 channels have been shown to mediate Ca^2+^ signaling, thereby regulating blood pressure bidirectionally (Chen et al., 2022).

### 3.2 Synthesis and transfer of lipids

It is worth noting that while the majority of enzymes involved in lipid synthesis are located in the membranes of the ER, there are also some present in mitochondrial membranes (Rowland and Voeltz, 2012; Petrungaro and Kornmann, 2019; Dong et al., 2024). MAMs are enriched with proteins related to lipid metabolisms, such as phosphatidylserine synthase 1/2 (PSS1/2), phosphatidylethanolamine N-methyltransferase (PEMT) 2, fatty acid CoA ligase 4, phosphatidylserine decarboxylase (PSD), caveolin-1 (CAV1), diacylglycerol O-acyltransferase, and Acyl-coenzyme A: cholesterol acyltransferase/sterol O-acyltransferase (ACAT/SOAT) (Luan et al., 2022; Wang et al., 2021; Zhang et al., 2023). The regulation of common cellular phospholipids, including phosphatidylcholine (PC), phosphatidylethanolamine (PE), and PS, is co-regulated by multiple proteins on MAMs. The PSS1/2 enzyme synthesizes PS on the ER, which is then transferred to the OMM through the ORP5/8-PTPIP51 complex. Subsequently, the PSD on the inner mitochondrial membrane (IMM) converts it to PE (Kimura and Kimura, 2021). PE is transferred from the mitochondria to the ER, where it undergoes PEMT2 methylation to generate PC (Rowland and Voeltz, 2012).

CAV1 is considered a crucial element of MAMs and is believed to play a role in regulating intracellular steroid and lipoprotein metabolism (Sala-Vila et al., 2016). ACAT is responsible for esterifying free cholesterol and storing cholesteryl esters in lipid droplets. It is worth noting that defects in ACAT function have been associated with atherosclerosis (Dove et al., 2006). Phosphatidic acid is synthesized in the ER and then translocated to the mitochondria for modification, ultimately resulting in the production of cardiolipin. This molecule has been shown to possess cardioprotective properties (Zhao and Wang, 2020). There is a strong association between PE and triacylglycerols with CVDs (Stegemann et al., 2014). The presence of excess lipids has been observed to create an intracellular environment that promotes Drp1 acetylation. As a result, there is an increase in its activity and mitochondrial translocation, which has been linked to cardiomyocyte dysfunction and death (Hu et al., 2020).

### 3.3 Mitochondrial dynamics

Mitochondria are organelles that undergo constant division and fusion, which is crucial for cellular function (Youle and van der Bliek, 2012). MFN1/2 and optic atrophy 1 (OPA1) are proteins involved in the regulation of mitochondrial fusion on MAMs, where MFN1/2 regulates the fusion of the OMM and OPA1 regulates the fusion of the IMM (Mishra and Chan, 2016). According to a study, a significant proportion of mitochondrial fission (84%) and fusion (59%) events appear to occur on MAMs, as observed through microscopy (Guo et al., 2018). Mitochondrial fission-mediated contraction takes place at the location of ER tubule-mitochondria contact (Friedman et al., 2011). Several proteins located in MAMs are involved in mitochondrial fission, including Drp1, Fis1, mitochondrial fission factor, and mitochondrial dynamics protein of 49 and 51 kDa, which form a protein complex that tightens mitochondria and initiates fission. Additionally, FUNDC1, inverted formin 2, syntaxin 17 (STX17), and ras analog in brain 32 are also involved (Ji et al., 2017; Luan et al., 2022).

Mitochondrial fusion prevents the loss of mitochondrial DNA and maintains mitochondrial protein synthesis, which is essential for proper mitochondrial function. Studies have shown that imbalances in protein activity may lead to mitochondrial disruption and increased damage to the cardiovascular system. For instance, excessive activity of Drp1 can lead to cardiac dysfunction due to excessive mitochondrial fragmentation (Hu et al., 2020). Metastasis-associated lung adenocarcinoma transcript 1 has been shown to inhibit mitochondrial dynamics and apoptosis through the miR-26b-5p/MFN1 pathway to improve cardiac microcirculation after myocardial infarction (Chen et al., 2021).

### 3.4 Autophagy

Autophagy is a biological process that is conserved in eukaryotic cells. It is regulated by autophagy-related genes and corresponding proteins (Feng et al., 2015). Studies have shown that autophagosomal membranes and many proteins related to autophagy are associated with MAMs, such as autophagosome markers autophagy-related (ATG) 5/14 (Hamasaki et al., 2013), and mechanistic target of rapamycin (mTOR) complex 2 (Colombi et al., 2011; Gomez-Suaga et al., 2017), a key inducer of autophagy. Furthermore, the VAPB-PTPIP51 complex on MAMs has been found to regulate autophagy (Wu et al., 2023). The depletion of MFN2 has been shown to significantly impair the generation of autophagy induced by starvation (Hailey et al., 2010). Autophagy is a process that selectively removes damaged mitochondria. PTEN-induced kinase 1, located in the damaged OMM, promotes Parkin translocation from the cytoplasm to the OMM, ubiquitinates the OMM protein MFN2 and the ion channel protein (VDAC), and thus promotes mitochondrial degradation (Gelmetti et al., 2017; Barazzuol et al., 2020). Additionally, hypoxia-induced FUNDC1, located in the OMM, has been reported to function as a mitochondrial receptor. It recruits autophagosomes and triggers mitochondrial degradation in response to hypoxia. Furthermore, FUNDC1 has been found to recruit Drp1 in MAMs, thereby promoting mitochondrial fission and autophagy (Wu et al., 2016).

Autophagic is considered to be crucial for maintaining the cardiovascular system. It has been suggested that excessive or insufficient autophagy may contribute to the development of CVDs (Gatica et al., 2015). The knockdown of essential autophagy genes, such as ATG5/7, can result in defective cardiac morphogenesis, particularly valve development and ventricular septum (Lee et al., 2014). In a sepsis model, it was observed that the heart increased autophagy by overexpressing Beclin-1. The inhibition of the mTOR signaling pathway was found to ameliorate septic cardiac dysfunction and alleviate inflammation and fibrosis. Conversely, knocking down Beclin-1 resulted in the opposite effect (Sun et al., 2018). Recent findings suggest that nitric oxide promotes mitochondrial autophagy, which is mediated by MCM8 and the E3 ubiquitin ligase TRIM21, and helps maintain normal coronary artery function (Lin et al., 2023).

### 3.5 Apoptosis

Apoptosis is a process of active cell death that is genetically controlled, also referred to as programmed cell death. Ca^2+^ transfer from the ER to the mitochondria plays a crucial role in apoptosis (Carpio et al., 2021). Ca^2+^ can easily pass through the OMM with the aid of ion channels, while the IMM is impermeable and Ca^2+^ can only enter through the mitochondrial Ca^2+^ uniporter, which has a relatively weak affinity for Ca^2+^ (Xu et al., 2020). Mitochondrial permeability transition pores (mPTP) can be formed when Ca^2+^ interacts with cyclophilin D and adenine nucleotide translocator. Excessive Ca^2+^ uptake by mitochondria can cause mPTP opening, which may lead to mitochondrial swelling and rupture of the OMM. This rupture can promote the release of apoptotic factors, including cytochrome C (Carraro et al., 2020). Several factors can impact Ca^2+^ levels in mitochondria, either directly or indirectly, by affecting the IP3R-GRP75-VDAC complex. For example, it has been observed that Akt, a serine-threonine protein kinase located in MAMs, phosphorylates IP3R. This leads to a reduction in Ca^2+^ release from the ER and a decrease in cellular sensitivity to Ca^2+^-dependent apoptosis (Marchi et al., 2017). Additionally, Bcl-2, a member of the Bcl-2 family located on MAMs, can bind to the central regulatory domain of IP3R to inhibit Ca^2+^ release. It can also indirectly inhibit IP3Rs by regulating their phosphorylation. In contrast, it has been observed that the proapoptotic proteins Bax and Bak of the Bcl-2 family regulate Ca^2+^ in the ER by binding to IP3R1 and replacing Bcl-2 (Rowland and Voeltz, 2012).

It is worth noting that apoptosis is closely related to the onset, progression, and regression of CVDs. A study reported that oxidative stress promotes PM2.5-induced cardiac injury in hyperlipidemic mice by activating apoptosis (Meng et al., 2022). Another study reported that inhibiting endothelial cell apoptosis can improve vascular dysfunction in vascular complications of type 2 diabetes (Su et al., 2018). Furthermore, a recent study has discovered that TMEM215 regulates Ca^2+^-mediated apoptosis by inhibiting MAMs, thereby modulating vascular pruning (Zhang et al., 2023).

### 3.6 Oxidative stress

MAMs are known to play a crucial role in regulating intracellular ROS and Ca^2+^. It has been reported that ER stress triggers Ca^2+^ release from the ER to mitochondria through MAMs. Conversely, ROS produced by mitochondria affects the ER, which can worsen ER stress and promote increased Ca^2+^ release. This, in turn, can lead to mitochondrial dysfunction and apoptosis or necrosis. Endoplasmic Reticulum oxidoreductase 1-alpha (Ero1α) is a critical regulator of protein folding and ER redox homeostasis. Its localization on MAMs is over 75% in oxygen-rich conditions (Gilady et al., 2010; Anelli et al., 2012). One study reported that homocysteine promotes Ero1α expression to produce H_2_O_2_ and further triggers ER oxidative stress (Wu et al., 2019). P66Shc, an oxidoreductase located on MAMs, interacts with cytochrome C to produce ROS (H_2_O_2_) as a signaling molecule for apoptosis (Giorgio et al., 2005). Another study found that Ca^2+^ induces the formation of H_2_O_2_ nanostructural domains at the MAMs, which modulate Ca^2+^ signaling and mitochondrial activity (Booth et al., 2016).

It is increasingly evident that oxidative stress contributes to the pathogenesis of CVDs (Senoner and Dichtl, 2019). For instance, one study reported that oxidative stress is associated with endothelial dysfunction in CVDs (Shaito et al., 2022). Another study reported that the promotion of PM2.5-induced cardiac injury in hyperlipidemic mice was associated with the activation of RyR2-regulated Ca^2+^ channels and apoptosis due to oxidative stress (Meng et al., 2022). Additionally, there are also studies showing that oxidative stress plays a crucial role in the development of HF and that its progression can be inhibited by anti-oxidative stress regulation of activating transcription factor 4 (Wang et al., 2022).

## 4 Mitochondria-associated endoplasmic reticulum membranes contribute to the mechanisms of several cardiovascular diseases

### 4.1 Myocardial infarction/Myocardial I/R injury

Acute myocardial infarction (AMI) is a significant cause of mortality globally. Hemodialysis is a vital tool to save the lives of AMI patients, but the resulting myocardial I/R injury is unavoidable. Studies have shown that the pathophysiologic mechanisms of I/R injury are associated with Ca^2+^ overload, ER stress, oxidative stress, mitochondrial autophagy, and apoptosis (He et al., 2022). Myocardial I/R injury is characterized by mPTP-mediated cell death. Mitochondrial Ca^2+^ overload triggers the opening of mPTP, resulting in mitochondrial swelling and the release of pro-apoptotic factors (Gao et al., 2020). One study suggested that mitochondrial autophagy could be a potential therapeutic target for treating I/R injury (Yang et al., 2019). After I/R injury, researchers found that liproxstatin-1 protected the heart by decreasing VDAC1 levels and mitochondrial ROS while inducing an increase in the antioxidant glutathione peroxidase 4 (Feng et al., 2019). Moreover, mitochondrial dynamics play an important role in myocardial I/R injury. As ischemia causes mitochondrial fragmentation, which is largely dependent on Drp1 and is associated with increased ROS and Ca^2+^ overload. Promoting mitochondrial fusion or inhibiting mitochondrial fragmentation may help protect the heart from I/R injury (Chen et al., 2023).

### 4.2 Diabetic cardiomyopathy

Diabetic cardiomyopathy (DCM) is a cardiomyopathy that is not related to hypertension or coronary artery disease. It is closely associated with a high incidence of HF and mortality in diabetic patients (Liu et al., 2024). The pathology of DCM is characterized by hypertrophy, necrosis, and apoptosis of cardiomyocytes, as well as myocardial interstitial fibrosis. The pathogenesis of DCM is a complex issue that involves multiple factors, such as insulin resistance, myocardial energy metabolism disorder, oxidative stress, inflammatory response, Ca^2+^ imbalance, autophagy, etc (Ritchie and Abel, 2020). Recent studies have suggested that there is a close association between MAMs and the development of DCM. Specifically, high glucose-induced aberrations of MAMs and mitochondrial dysfunction have been implicated in the pathogenesis of cardiomyopathy (Salin Raj et al., 2023). One study showed that elevated glucose levels were associated with increased levels of FUNDC1, IP3R2, and MAMs, resulting in mitochondrial dysfunction and increased Ca^2+^ transfer (Wu et al., 2019). Additionally, an *in vitro* study found that cardiomyocytes cultured in high-glucose and high-fat media displayed excessive mitochondrial fission and low MFN2 expression. The restoration of MFN2 was found to have a positive impact on mitochondrial membrane potential, reducing mitochondrial oxidative stress and alleviating mitochondrial dysfunction in cardiomyocytes (Hu et al., 2019). Furthermore, it was observed that PERK deficiency exhibited a protective effect against high glucose-induced cardiomyocyte apoptosis by reducing ROS-mediated activation of the PERK signaling pathway, which causes ER stress-induced apoptosis (Liu et al., 2013).

### 4.3 Cardiac hypertrophy/HF

Cardiac hypertrophy is a significant paleopathology of HF. According to studies, TRPV1 has been found to promote the formation of MAMs and stabilize mitochondrial function through the AMP-activated protein kinase/MFN2 pathway in cardiomyocytes. This has been shown to effectively prevent stress-induced cardiac hypertrophy (Wang et al., 2022). HF is a condition that can occur as a result of various CVDs. It is characterized by structural and/or functional abnormalities of the heart that can lead to increased intracardiac pressure and/or insufficient cardiac output, both at rest and during exercise (McDonagh et al., 2021). Previous studies have shown that Ca^2+^ homeostasis and mitochondrial function play a key role in cardiac remodeling and HF (Chaanine, 2021). FUNDC1 is localized to MAMs and regulates Ca^2+^ release from the ER by binding to the ER-resident protein IP3R2. Disruption of these interactions can lead to reduced mitochondrial and cytoplasmic Ca^2+^, which may result in aberrant mitochondrial fission and dysfunction. Ultimately, this may contribute to cardiac dysfunction and HF (Wu et al., 2017). LonP1 is a protease that is known to be localized in the MAMs. Studies have shown that LonP1 deficiency can damage the integrity of MAMs and mitochondrial fusion, which may lead to the activation of the unfolded protein response within the ER. This could potentially result in remodeling of the heart and eventual progression to HF (Li et al., 2023).

### 4.4 Myocardial injury

Recently, MAMs have been shown to have an important role in myocardial injury. Specifically, studies have demonstrated that dibutyl phthalate can induce ER stress in cardiomyocytes, leading to an increase in MAMs and subsequent mitochondrial damage caused by abnormal Ca^2+^ transfer. Furthermore, the production of mitochondrial ROS can activate the NLRP3 inflammasome and pyroptosis in cardiomyocytes, which can ultimately result in cardiac injury (Li et al., 2023). In a study, sheep were used as research subjects and were administered Mo and Cd through continuous gavage for 50 days (Peng et al., 2023). The study found that this led to cardiac autophagy and damage to myocardial morphology in sheep, which was attributed to ER stress, mitochondrial dysfunction, and structural disruption of MAMs. According to a recent study, the formation of the MAMs protein FUNDC1 and intracellular Ca^2+^ levels are regulated by sepsis through the IL-6/STAT3 pathway, which can result in mitochondrial disruption, expression of mitochondrial autophagy proteins, and ROS production, ultimately leading to myocardial dysfunction (Jiang et al., 2022). Moreover, ATG, a scaffolding molecule located on MAMs, may play a significant role in obesity-induced cardiomyopathy. Recently study suggested that STX17 may contribute to cardiac damage associated with obesity through the formation of MAMs, which can lead to mitochondrial Ca^2+^ overload, O_2_- accumulation, and lipid peroxidation (Xu et al., 2023). Additionally, long-term consumption of diets high in fat and sucrose may cause abnormal lipid accumulation in both endothelial and myocardial cells in individuals with metabolic syndrome due to induced CAV1 expression. Lipid accumulation and lipotoxicity may have an impact on the destruction of MAMs and mitochondrial remodeling in cardiomyocytes, which could potentially lead to cardiomyocyte apoptosis, cardiac dysfunction, and remodeling (Liu et al., 2023).

### 4.5 Other CVDs

Recently, it has been found that MAMs have a crucial role in the proliferation of VSMCs. Under hypoxic conditions, the downregulation of the Nogo-B receptor leads to the destruction of MAMs. This, in turn, enhances the phosphorylation of IP3R3 via pAkt and promotes VSMCs proliferation (Yang D. et al., 2019). Furthermore, it has been noted that PEMT located in MAMs plays a significant role in the regulation of phospholipid metabolism. Another study reported that diet-induced atherosclerosis can be prevented by PEMT knockdown (Li et al., 2023). MAMs are significantly involved in age-related CVDs. Specifically, during cardiac aging, the integrity of MAM contact sites was disrupted and the dynamic balance of Ca^2+^ was dysregulated. Additionally, the expression level of Cisd2, a longevity gene located on MAMs, was observed to decrease with cardiac aging. It is worth noting that high levels of Cisd2 may have a positive effect on delaying cardiac aging and improving age-related cardiac dysfunction (Yeh et al., 2020). Recent studies have suggested that TMEM215 may protect endothelial cells from apoptosis by inhibiting BIK-mediated Ca^2+^ flow in MAMs by facilitating BiP/BIK complex interactions. The reduction of TMEM215 resulted in notable changes, including an increase in the number of MAMs, a decrease in the distance between the OMM and the ER membrane, and an elevation in mitochondrial Ca^2+^ and Cyto C levels. These changes are known to help with the regulation of vascular pruning (Zhang et al., 2023).

## 5 Mitochondria-associated endoplasmic reticulum membranes as potential targets for cardiovascular diseases

Scholars have proposed and explored the possibility of MAMs as potential new targets for the prevention or treatment of CVDs (Table 1). One reported that hyperglycemia was found to increase interactions between mitochondria and ER, as well as mitochondrial apoptosis (Yang et al., 2017). However, exogenous H_2_S was observed to reduce mitochondrial apoptotic proteins, cytochrome c, mPTP opening, and MFN2 expression, which had a cardioprotective effect. Additionally, researchers discovered that the herbal formula Yiqi Huoxue reduced the Sigma-1 receptor (a chaperone protein on MAMs) and increased IP3R2 expression in infarcted rats, which helped prevent cardiomyocyte hypertrophy (Lou et al., 2021). Another study reported that FeA was found to attenuate MAM alterations induced by high glucose and ameliorate cardiomyopathy in diabetic rats (Salin Raj et al., 2023). This was achieved by activating the pro-apoptotic protein PACS2/IP3R2/FUNDC1/VDAC1 pathway. A recent study found that the herbal extract Luteolin may attenuate palmitic acid-induced lipotoxic myocardial injury via the ER stress-mitochondrial apoptosis pathway (Xiang et al., 2023). However, these pieces of evidence have only been attempted before clinical trials, and further validation and clinical translation are needed in the future.

**TABLE 1:** Summary of research mechanisms based on MAMs for treating CVDs.

Study	Intervention	Treatment of diseases	Functions and mechanisms	Outcomes
Yang et al. (2017)	Exogenous H_2_S	DCM	The expression of mitochondrial apoptotic proteins, cytochrome c, and mPTP opening was decreased following treatment with exogenous H_2_S, thus reducing the expression of ER stress sensors and apoptotic rates in cardiac tissue of DCM and cultured H9C2 cells	Hyperglycemic stimulation of ER interactions and mitochondrial apoptotic pathways were inhibited by exogenous H_2_S through the regulation of MFN2 expression
Lou et al. (2021)	Yiqi Huoxue (YQHX) prescription	Myocardial infarction-induced HF	YQHX was revealed to attenuate the decreased expression of the Sig1R and increase the expression of IP3R2 in myocardial infarction rats. Further, YQHX prevented cell hypertrophy and normalized the decreased ATP content	The cardioprotective effect of YQHX is mediated through the Sig1R located on the MAMs
Salin Raj et al. (2023)	FeA	DCM	High glucose conditions caused the upregulation of MAMs formation via PACS2, IP3R2, FUNDC1, and VDAC1 and decreased mitochondrial biogenesis, fusion and oxidative phosphorylation. FeA effectively prevented the high glucose-induced MAMs alterations and associated cellular anomalies both in vitro and in vivo	MAMs could be explored as a potential target to treat diabetic cardiomyopathy. Also, the FeA could be an attractive nutraceutical agent for diabetic cardiomyopathy
Xiang et al. (2023)	Luteolin	Lipotoxic Myocardial Injury	Palmitic acid (PA, 0.2 mmol/L) stimulation of aberrant protein expressions was significantly reversed after treatment with Luteolin, which meanwhile reduced the level of PA-induced oxidative stress in H9C2 cardiomyocytes, alleviated the degree of apoptosis, and reversed the expression of proteins related to the endoplasmic reticulum stress-mito-chondrial apoptosis pathway under the influence of PA.	Luteolin can alleviate PA-induced lipotoxic myocardial injury, and this process may be related to the endoplasmic reticulum stress-mi-tochondrial apoptosis pathway

CVDs, Cardiovascular diseases; DCM, diabetic cardiomyopathy; ER, endoplasmic reticulum; FUNDC1, FUN14 domain-containing 1; HF, heart failure; IP3R, The inositol 1,4,5-trisphosphate receptor; MAMs, Mitochondria-associated endoplasmic reticulum membranes; MFN2, Mitofusin 2; mPTP, mitochondrial permeability transition pores; PA, palmitic acid; PACS2, Phosphofurin acidic cluster sorting protein 2; Sig1R, Sigma 1 receptor; VDAC1, Voltage-dependent anion channel 1; YQHX, yiqi huoxue.

## 6 Summary and outlook

**FIGURE 1:**
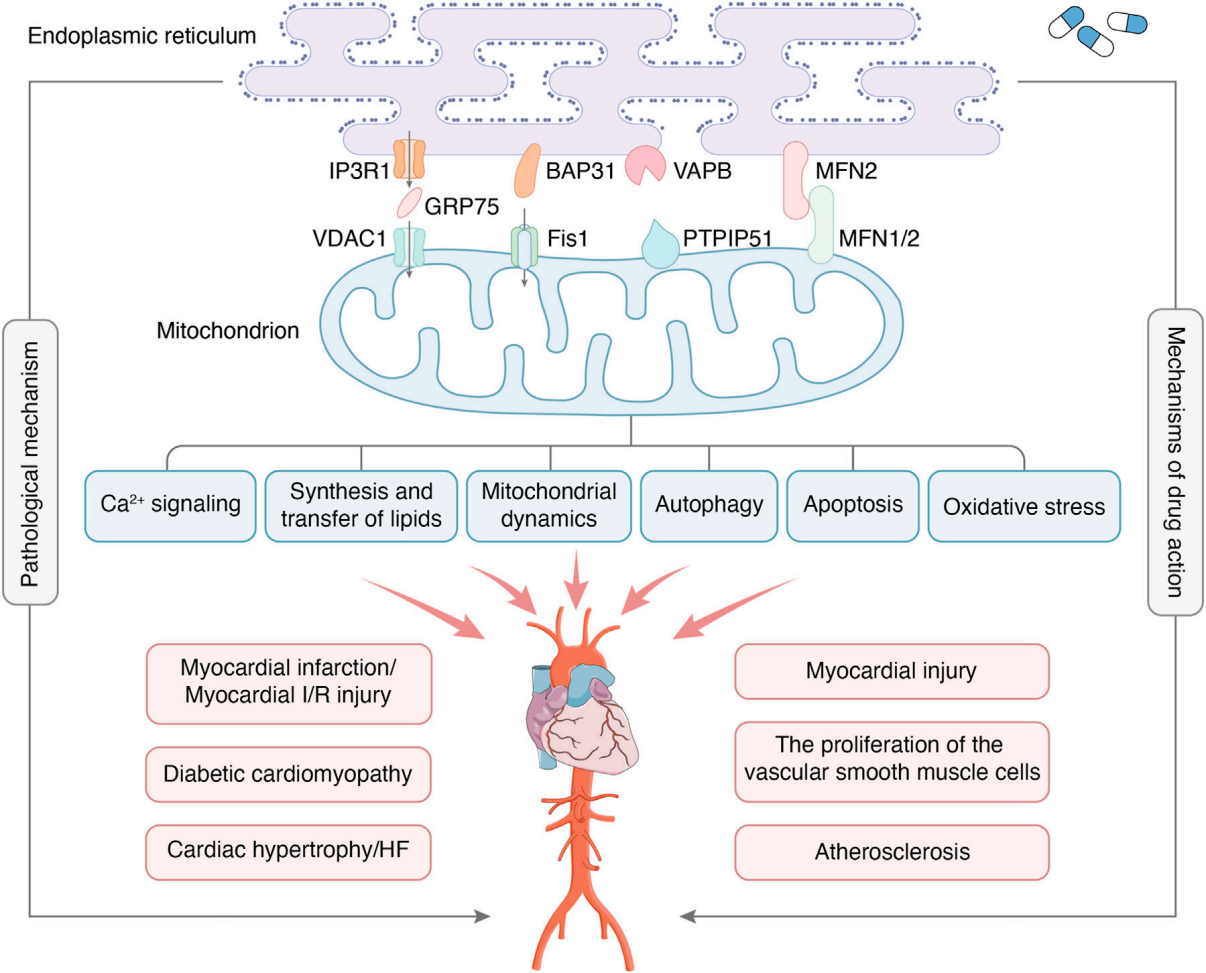
MAMs are regions where the OMM and certain areas of the ER membrane overlap without membrane fusion, which enables communication between organelles and bi-directionally regulates cellular functions through connectivity proteins. The major protein complexes on MAMs include IP3R1-GRP75-VDAC1, BAP31-Fis1, VAPB-PTPIP51, MFN2-MFN1/2, etc. The main functions of MAMs include Ca^2+^ signaling, synthesis and transfer of lipids, mitochondrial dynamics, autophagy, apoptosis, oxidative stress, etc. MAMs are crucial in developing CVDs, including myocardial infarction, myocardial I/R injury, diabetic cardiomyopathy, etc. At the same time, MAMs have also become the target of drug therapy for CVDs.

MAMs, physical connections between the ER and mitochondria, have been found to play a crucial role in regulating cellular metabolism in both physiological and pathological states. This study provides an overview of the structure and function of MAMs, as well as their mechanism of action in CVDs. Additionally, the clinical significance of MAM-based interventions is discussed. A comprehensive understanding of the regulatory mechanisms of the MAMs in CVDs is important for identifying new therapeutic targets for the prevention or treatment of CVDs (Figure 1). For instance, while ketogenic diets are popular among diabetic patients, it is important to consider their safety. A study found that the ketogenic diet improved metabolic markers and reduced MAMs in patients (Tao et al., 2021). However, the study also points out that the ketogenic diet promoted cardiac fibrosis by inhibiting mitochondrial and T-regulatory cell function. Similarly, Sor has toxic side effects on the heart during tumor treatment and may affect the continuation of treatment. Excitingly, researchers found Sor appears to mediate the downregulation of MFN2 in a toxic-dependent manner, which leads to over-formation of MAMs and Ca^2+^ overload (Song et al., 2022). This, in turn, causes necrotic apoptosis in cardiomyocytes. Furthermore, overexpression of MFN2 inhibits cardiomyocyte necrotic apoptosis without interfering with the antitumor effect. This approach enables the potential for extended treatment of tumors. In addition, it should be noted that exercise preconditioning has the potential to regulate MAMs, which may contribute to a protective effect on the heart (Lv et al., 2022).

Although the structure and function of MAMs and their relationship to CVDs have been recognized, there are still some unknown areas waiting to be explored by humans. Firstly, it is important to investigate whether MAMs have the same structure and function in different tissues of the cardiovascular system. If not, what are the key mechanisms by which they function? Secondly, it is necessary to examine the effects of age, gender, and behavioral factors such as late nights, alcohol consumption, and lack of exercise on MAMs. Do these factors contribute to CVDs through MAMs? Finally, as an important signaling platform, can MAMs play a preventive role as early markers of CVDs? In conclusion, a deeper understanding of the MAMs platform is needed to propose optimal strategies for the prevention and treatment of CVDs.

## Figures and Tables

**Table:** 

**ACAT/SOAT**	Acyl-coenzyme A: cholesterol acyltransferase/sterol O-acyltransferase
**AMI**	Acute myocardial infarction
**ATG**	Autophagy-related
**BAP31**	B-cell receptor-associated protein 31
**BiP**	Binding immunoglobulin protein
**CAV1**	Caveolin-1
**CVDs**	Cardiovascular diseases
**DCM**	Diabetic cardiomyopathy
**Drp1**	Dynamin-related protein 1
**ER**	Endoplasmic reticulum
**Ero1α**	Endoplasmic Reticulum oxidoreductase 1-alpha
**Fis1**	Fission protein 1
**FUNDC1**	FUN14 domain-containing 1
**GRP75**	Glucose-regulated protein 75
**HF**	Heart failure
**I/R**	Ischemia-reperfusion
**IMM**	Inner mitochondrial membrane
**IP3**	Inositol trisphosphate
**IP3R**	The inositol 1,4,5-trisphosphate receptor
**LonP1**	Lon protease 1
**MAMs**	Mitochondria-associated endoplasmic reticulum membranes
**MERCs**	Mitochondria-endoplasmic reticulum contacts
**MFN1/2**	Mitofusin 1 and 2
**mPTP**	Mitochondrial permeability transition pores
**mTOR**	Mechanistic target of rapamycin
**OMM**	Outer mitochondrial membrane
**OPA1**	Optic atrophy 1
**PA**	Palmitic acid
**PACS2**	Phosphofurin acidic cluster sorting protein 2
**PC**	Phosphatidylcholine
**PE**	Phosphatidylethanolamine
**PEMT**	Phosphatidylethanolamine N-methyltransferase
**PERK**	Protein kinase RNA-like endoplasmic reticulum kinase
**PS**	Phosphatidylserine
**PSD**	Phosphatidylserine decarboxylase
**PSS1/2**	Phosphatidylserine synthase 1/2
**PTPIP51**	Protein tyrosine phosphatase interacting protein 51
**ROS**	Reactive oxygen species
**RyR**	Ryanodine receptor
**Sig1R**	Sigma 1 receptor
**STX17**	Syntaxin 17
**TMEM215**	The transmembrane protein 215
**TRIM21**	Tripartite motif containing 21
**TRPV**	Transient receptor potential vanilloid
**VAPB**	Vesicle-associated membrane protein-associated protein B
**VDAC1**	Voltage-dependent anion channel 1
**VSMCs**	Vascular smooth muscle cells
